# Analytical Capacitance Model for Carbon Nanotube Field-Effect Transistors Including Interface-Trap Effects

**DOI:** 10.3390/nano15080604

**Published:** 2025-04-14

**Authors:** Bin Zhou, Rui Zhan, Zilin Teng, Yiheng Xue, Xiaoyan Hu, Jianhua Jiang, Panpan Zhang

**Affiliations:** 1 School of Integrated Circuits, Beijing University of Posts and Telecommunications, Beijing 100876, China; bynn@bupt.edu.cn (B.Z.); xyh100083@bupt.edu.cn (Y.X.); 2 National Key Laboratory of Integrated Circuits and Microsystems, Beijing 100042, China; huxiaoyan@cetc.com.cn; 3 Key Laboratory for the Physics and Chemistry of Nanodevices, Center for Carbon-Based Electronics, School of Electronics, Peking University, Beijing 100871, China

**Keywords:** carbon nanotube field-effect transistors (CNTFETs), interface traps, quantum capacitance, small-signal model, compact model

## Abstract

The creation of carbon nanotubes has sparked a paradigm shift in the post-silicon era because of their decent electronic and optical properties. However, interface traps pose an obstacle in the realization of high-performance carbon nanotube field-effect transistors (CNTFETs). Herein, we systematically investigate the C−V characteristics of CNTFETs and propose a small-signal equivalent model to decouple the effects arising from interface traps. Moreover, intrinsic parameters associated with interface traps can be feasibly extracted using this approach. An analytical capacitance model is further developed to be incorporated into the well-established CNTFET virtual source model, and excellent agreement has been achieved between our simulation and the measured results of the as-fabricated MOSCAP. The models developed here help to gain insight into the physical properties of high-κ dielectric interface traps subjected to different processes and inform strategies to achieve high-performance CNTFETs.

## 1. Introduction

Data-intensive computing applications have driven the continuous reduction of critical dimensions to improve transistor density. The shrinking bottleneck of silicon technology has stimulated research interest into low-dimensional materials, such as carbon nanotubes (CNTs), due to their decent electrical properties. CNTs have demonstrated their superiority in implementing high-performance field-effect transistors (FETs) and integrated circuits over the past two decades [1,2,3,4,5,6,7,8,9]. However, the presence of traps at the dielectric and channel interface significantly erodes gate control and reduces device reliability, thus preventing CNTs from unleashing their full potential in practical applications. Extensive efforts have been devoted to investigate the physical origin of substantial interface traps of carbon nanotube field-effect transistors (CNTFETs), which should be ascribed to the perfect surface of CNTs, which are free of dangling bonds and are unable to provide nucleation sites for high-κ dielectrics (e.g., HfO_2_). Moreover, optimization strategies for the suppression of interface traps have also been broadly explored [10,11,12,13,14,15,16,17,18]. To enable a comprehensive evaluation of the interface trap effects at both the device and circuit levels for optimization strategies, a compact CNTFET model that accurately incorporates these traps is essential [19,20,21,22,23,24,25,26]. Although the virtual-source model of CNTFETs (VSCNTFET) published by Stanford University is well-established and widely used in the research community, the impact of interface traps is still lacking [27,28,29], potentially limiting its accuracy for frequency-dependent or transient circuit simulations where interface states are influential.

This study proposes a small-signal equivalent circuit model for CNTFETs that incorporates the effects of interface traps, enabling the agile extraction of crucial gate capacitance parameters such as quantum capacitance, interface trap density, and trap-time constants to capture and emit carriers. Based on these extracted parameters, analytical expressions for quantum capacitance and interface trap capacitance can be well established. Subsequently, the VSCNFET model is upgraded to include the impact of interface traps on both C−V and I−V characteristics, effectively capturing the frequency dispersion observed in the C−V characteristics of CNT-based metal-oxide-semiconductor capacitors (MOSCAP). Finally, the validity of this compact model is demonstrated by showing excellent agreement with measured C−V characteristics obtained from the fabricated CNTFET MOSCAP devices.

The manuscript is organized as follows: First, we present the CNT MOSCAP structure and its small-signal model to emulate the behavior of the total−gate capacitance. Subsequently, the proposed small-signal model is used to analyze the measured C−V data, along with a comparison with the conventional high-frequency/low-frequency approach. Next, the paper focuses on developing analytical models for each component of the total-gate capacitance and updating the VSCNFET compact model to incorporate these effects. Finally, we demonstrate the accuracy of the model by comparing it with the measured C−V characteristics of the fabricated CNTFET MOSCAP devices.

## 2. Theoretical Concept

**Figure 1:**
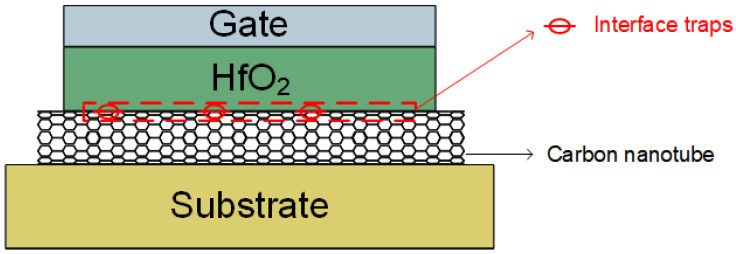
CNT-Based MOS Capacitor Structure.

**Figure 2:**
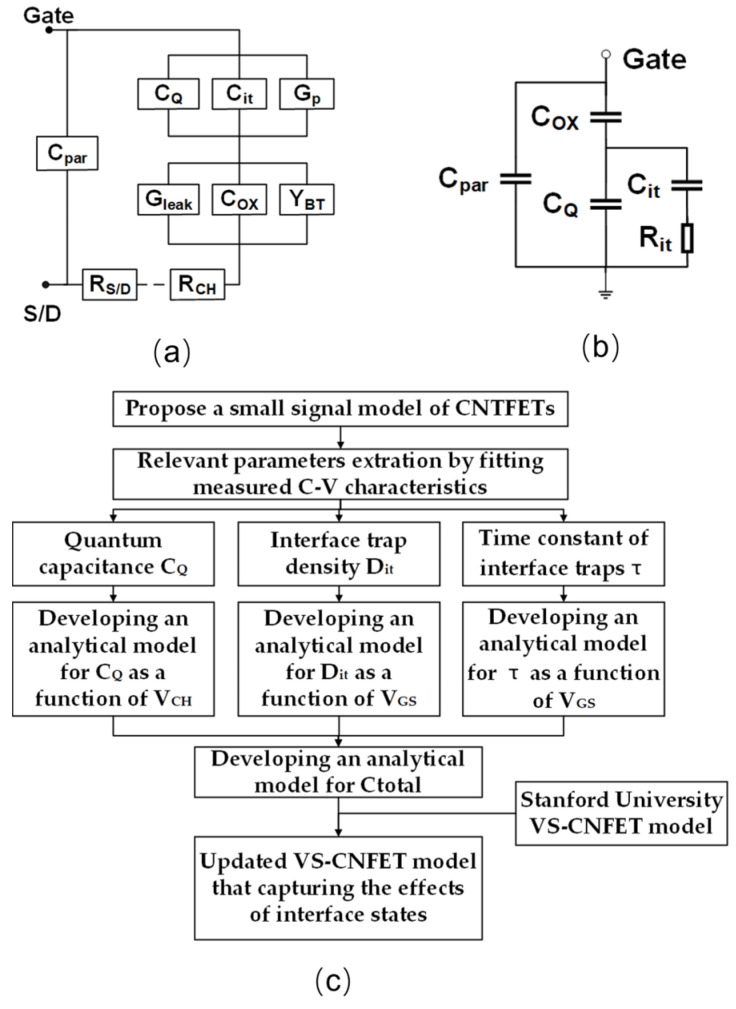
Gate composition of the CNTFET device and modelling flowchart. (**a**) Transmission line network between the gate and source/drain. (**b**) A simplified equivalent gate capacitance model of interest in this work. (**c**) The flowchart showcasing the process of developing the updated VS-CNFET model that incorporates the effects of interface states.

Figure 1 illustrates the cross section of CNT MOS structure that we investigate in this work. The device utilizes an aligned carbon nanotube array as the channel of L = 100 nm and W = 1 µm, with 9 nmHfO_2_/4 nm Ti serving as the gate stack. Figure 2a illustrates the full gate composition of CNTFET, encompassing effects from interface traps, quantum confinement, fringe-electric field, contact resistance, and so forth [30]. This research primarily focuses on the analysis of quantum capacitance and interface-trap effects, which have the most significant impact, with a simplified equivalent circuit shown in Figure 2b. Extensive efforts have been devoted to investigate the quantum confinement effect in CNTs, which results in a remarkable quantum capacitance that cannot be omitted [31]. Fundamentally, this quantum capacitance arises from the limited density of states available for charge carriers; therefore, it is closely related to the CNT’s electronic structure, specifically its diameter and chirality. Additionally, considerable interface traps exist at the heterogeneous interface between the channel and atomic layer deposition (ALD)-grown high-κ dielectric HfO_2_, which trap channel carriers and deteriorate the gate control through an equivalent network of capacitors and resistors in series, denoted as C_it_ and R_it_, respectively [32]. The interface-trap density (Dit) determines the capability of interface traps to capture carriers, and the time constant of interface traps (τ) represents the characteristic time needed for these traps to exchange carriers with the CNT bands, based on the Shockley−Read−Hall (SRH) recombination process. When the period (inverse of frequency) of the small-signal voltage is shorter than the time constant (τ), the interface traps gradually become unable to respond to the signal, leading to a reduction in the number of captured carriers.

## 3. Results and Discussion

Figure 2c sheds light on the overall flow to develop the gate capacitance model. Generally, relevant parameters were first extracted from the measured C−V characteristics and then fed into the proposed analytical gate capacitance model.

**Figure 3:**
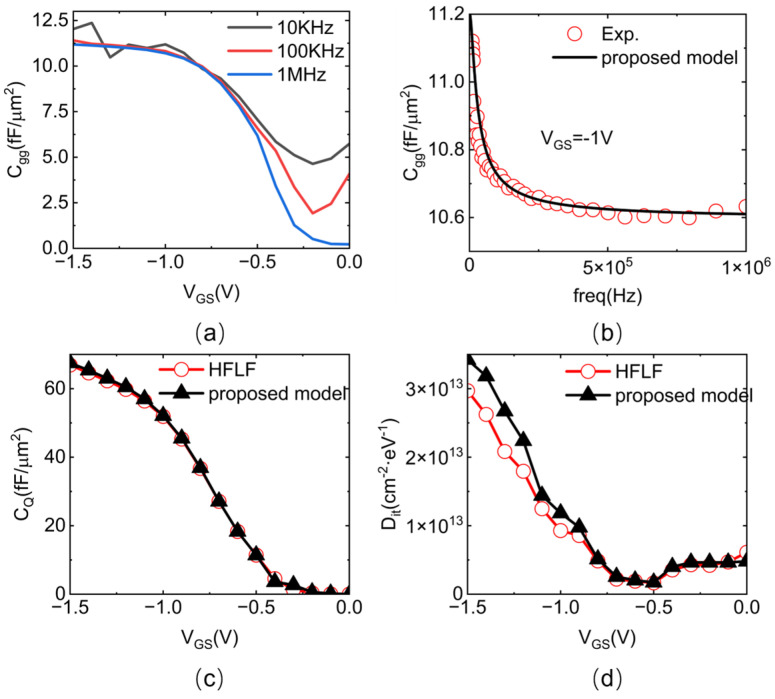
C−V characteristics and extracted parameters. (**a**) As-measured multifrequency C−V curves. (**b**) Illustration of the proposed model fitting method at *V_GS_* = −1 V. (**c**) Comparison of the *C_Q_* parameter values obtained by extraction using the HFLF method and model fitting. (**d**) Comparison of *D_it_* parameter values obtained by extraction using the HFLF method and model fitting.

The measured C−V characteristics of a CNT MOSCAP were borrowed from the previous work by our group with normalized results, as shown in Figure 3a. More details about device fabrication and measurement can be found in Reference [33]. Prior to the analysis, the values of the two bias- and frequency-independent capacitance components, *C_OX_* and *C_par_*, were first determined using an empirical approach [34]. *C_OX_* is approximately equal to 1.2 times the maximum value of the high-frequency C−V characteristic, which is 13.5 fF/um^2^ in this work. *C_par_* can be determined as the minimum value of high-frequency C−V characteristics, which reads as 0.7 fF/µm^2^ from Figure 3a (including the parasitic capacitance at both the source and drain terminals, which are eliminated in the following analysis).

As shown in Figure 2b, the gate oxide capacitance (*C_OX_*) is in series with the parallel combination of the quantum capacitance (*C_Q_*) and interface-trap effects, denoted as a cascade of capacitance *C_it_* and resistance *R_it_*. Note that the RC equivalent sub-circuit characterizes the frequency dependence of interface traps, where *C_it_* = *q*^2^ × *D_it_* and *R_it_ × C_it_ = τ*^−1^. Therefore, *C_total_* of the gate capacitance can be expressed as Equation (1) [35]:

(1) $$\frac{1}{C_{total}}=\frac{1}{C_{OX}}+\frac{1}{\left(C_{Q}+C_{it}\right)}\;=\;\frac{1}{C_{OX}}+\frac{1}{\left[C_{Q}+q^{2}D_{it}{(2\pi f\tau )}^{-1}\arctan(2\pi f)\right]}$$

where *q* represents the elementary charge; *D_it_* represents the interface trap density; *τ* represents the time constant; *q*^2^*D_it_*(2*fπτ*)^−1^arctan(2*πf*) is a compact mathematical representation of the RC sub-circuit, as above mentioned; and *f* is the frequency of the small-signal AC voltage applied to the device.

### 3.1. Parameter Extraction

Generally, several methods for evaluating the effects of the interface trap include the subthreshold swing (SS) method, the high-frequency and low-frequency capacitance (HFLF) method, and the gate conductance (*G_p_*) method. The SS method is the easiest method, but it sacrifices accuracy, where the interface traps are equivalent into a capacitor in series with the gate oxide capacitance. The *G_p_* method, which involves measuring the gate impedance, provides sufficient accuracy but is complicated to implement. The HFLF method, implemented as a reference in this work, serves as the tradeoff between the. SS and *G_p_* methods, which provides both acceptable accuracy and feasibility of operation.

Thus, we first extract *D_it_* and *C_Q_* through the HFLF method based on the C−V curves presented in Figure 3a. In practice, *C_Q_* and *D_it_* are typically calculated using Equations (2) and (3) [36,37]:

(2) $$C_{Q}=\frac{C_{HF}\times C_{OX}}{C_{OX}-C_{HF}}$$

(3) $$D_{it}=\frac{C_{OX}}{Aq^{2}}\left(\frac{C_{LF}}{C_{OX}-C_{LF}}-\frac{C_{HF}}{C_{OX}-C_{HF}}\right)$$

where *A* is the effective channel area of CNTFET and *C_LF_* and *C_HF_* are the specific capacitance measured at 10 kHz and 1 MHz, respectively.

We first calibrated the proposed total-gate capacitance model with the experimental results of capacitance versus frequency. Generally, *C_total_* decreases with increasing frequency because interface traps cannot completely respond to high frequencies. Ultimately, *C_total_* saturates at a value determined by (1/*C_OX_* + 1/*C_Q_*). The fitting procedure was conducted iteratively until our proposed model achieved excellent agreement with the experimental data. Several parameters could eventually be extracted, e.g., the quantum capacitance (*C_Q_*), interface trap density (*D_it_*), and time constant (*τ*).

As shown in Figure 3c,d, the extracted *C_Q_* and *D_it_* versus the gate-source voltage using our proposed model coincided well with the results obtained by the well-settled HFLF approach, implying the validity of our model. Note that our methodology further enables the extraction of the time constant (τ) through the measured C−V characteristics—a crucial dynamic parameter inaccessible in the conventional HFLF method.

### 3.2. Analytical Model Development

In order to implement the proposed total capacitance network in the compact model of CNTFETs, accurate analytical expressions for each component are essential.

For the sake of simplicity, the aligned CNT array could be assumed to be similar to the two-dimensional material, using established 2D quantum capacitance models to bypass 1D inter-tube coupling calculations. In this 2D equivalence framework, key parameters like density of states and carrier concentration represent statistical averaging across the CNT array.

The quantum capacitance *C_Q_* can thus be determined theoretically from the Fermi−Dirac distribution and the density of states (DOS) of 2D materials, the explicit form of which is expressed as Equations (4) and (5) [38]:

(4) $$C_{Q}=q^{2}g_{2D}\left[1+\exp(\frac{E_{g}}{2k_{B}T})/2\cosh\left(\frac{qV_{CH}}{k_{B}T}\right)\right]$$

(5) $$g_{2D}=\frac{g_{s}g_{v}m^{*}}{2\pi \hbar^{2}}$$

where *E_g_* is the band gap of CNT, *V_CH_* is the surface potential of the CNTFET, *g_s_* = 2 is the spin degeneracy factor, *g_v_* = 2 is the valley degeneracy factor, *ħ* is the reduced Plank constant, and m* is the effective mass for electrons of the carbon nanotube. Furthermore, the channel potential, *V_CH_*, is described by the following empirical model, as shown in Equation (6) [39]:

(6) $$V_{CH}=S_{Fn}\left[V_{F\mathrm{nt}}\ln\left(1+\exp\left(\frac{V_{gt}}{V_{Fnt}}\right)\right)-\right.\left.V_{FnB}\ln\left(1+\exp\left(\frac{V_{gt}-\beta_{Fn}V_{DS}}{V_{FnB}}\right)\right)\right]$$

where *V_gt_* = *V_GS_* − *V_th_* is the gate overdrive voltage; parameter *S_Fn_* allows for adjusting the slope, i.e., the proportionality with *V_GS_*_;_, the voltage parameters *V_Fnt_* and *V_FnB_* determine the smoothness of the transitions; parameter *β_Fn_* allows for modeling the deviation after the transition from Boltzmann to Fermi statistics.

**Figure 4:**
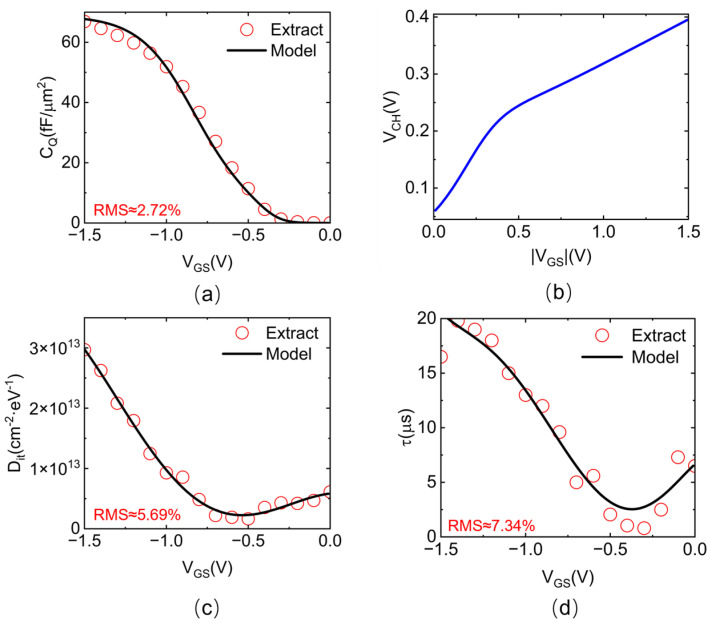
The validation of the proposed analytical models for each parameter presented in the *C_total_* expression. (**a**) Fitting results of the analytical model for C_Q_ versus extracted values from the measured C−V characteristics. (**b**) The extracted surface potential for CNTFETs. (**c**) Fitting results of the analytical model for *D_it_* versus extracted values from the measured C−V characteristics. (**d**) Fitting results of the analytical model for the time constant τ versus extracted values from the measured C−V characteristics.

Recognizing that the virtual source model parameters depend solely on terminal voltages (*V_GS_* and *V_DS_*), both the interface trap density and carrier lifetime can be accurately represented as functions of *V_GS_* in our experimental data. To this end, we utilize a Fourier function to model the interface trap density (*D_it_*) and a polynomial function to model the carrier lifetime (*τ*), respectively.

(7) $$D_{it}=a_{0}+a_{1}\cos(wV_{GS})+b_{1}\sin(wV_{GS})+a_{2}\cos(2wV_{GS})+b_{2}\sin(2wV_{GS})$$

(8) $$\tau =p_{1}{V_{GS}}^{5}+p_{2}{V_{GS}}^{4}+p_{3}{V_{GS}}^{3}+p_{4}{V_{GS}}^{2}+p_{5}V_{GS}+p_{6}$$

where *a*_0~2_, *b*_1~2_, *ω*, and *p*_1~6_ are empirical fitting parameters extracted through nonlinear least-squares regression using MATLAB (2021b) Curve Fitting Toolbox (cftool). It should be noted that the fifth-order polynomial function was selected for its ability to achieve a high fitting accuracy, demonstrating a root mean square (RMS) error of less than 8%. Figure 4 shows the fitting results of each gate component, exhibiting the accuracy of our proposed models.

**Figure 5:**
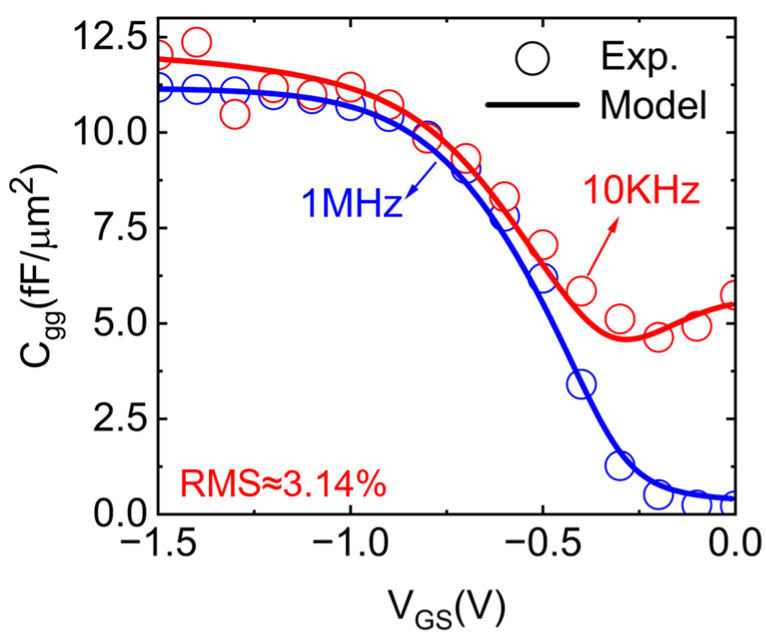
Comparison of the comprehensive gate model with experimental 10 kHz and 1 MHz CV data.

We finally arrived at an analytical expression for the total capacitance with each item being figured out. As shown in Figure 5, the proposed *C_total_* model accurately captured the frequency-dependent dispersion characteristics of the CNTFET gate. The model exhibited good agreement with the C−V characteristics of the fabricated MOSCAP, confirming the accuracy of the analytical model.

### 3.3. VSCNFET Model Enhancement

**Figure 6:**
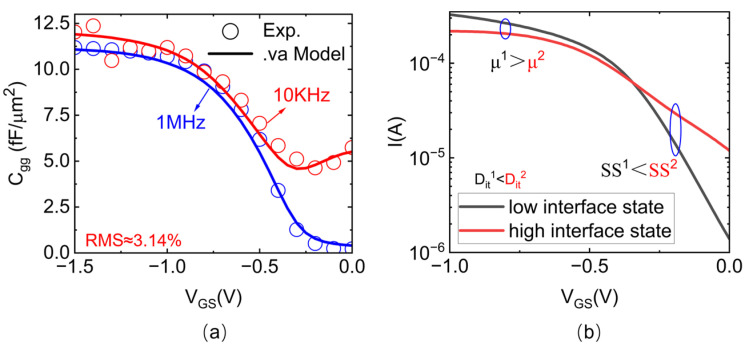
The characteristics of the updated VS-CNFET model. (**a**) Comparison of C−V characteristics between the updated VS-CNFET model and experimental data at 10 kHz and 1 MHz, respectively. (**b**) Influence of interface traps on the transfer characteristics of CNTFETs projected by the model.

Subsequently, we extended the Stanford University VS-CNFET model [27,28] to develop a compact CNTFET model that accounts for interface- trap effects, as derived in the above sections. As shown in Figure 6a, the updated VS-CNFET model accurately fits the measured C−V characteristics of the fabricated CNTFET MOSCAP. Additionally, the model incorporates a *V_DS_*-dependent source-drain charge partitioning, transitioning from a 1:1 ratio at low V_DS_ to a 3:2 ratio at high *V_DS_*. Furthermore, the model accounts for the influence of the interface trap on the I−V characteristics, specifically the sub-threshold swing and effective mobility, by considering the carrier allocation between the interface trap capacitance and the channel capacitance. Further, the proposed CNTFET capacitance model exhibits strong generality across device architectures. The gate capacitance network topology is compatible with most common CNTFET configurations, while key parameters such as *C_OX_*, *C_Q_*, *D_it_*, and τ are empirically modeled from the measured C−V characteristics without relying on specific device geometries. This framework enables consistent characterization of the. interface-trap effects in CNTFETs with varying structures and dimensions, as validated through both large-area MOSCAP measurements and scaled device analyses.

## 4. Conclusions

In this work, we propose a small-signal network for the total-gate capacitance of CNTFETs to interpret the influences from interface-trap states and quantum confinement effects. With several extracted parameters from the measured C−V characteristics, explicit expressions for each component of the network have also been determined, successfully capturing the observed frequency dispersion features. Further, we upgrade the well-established virtual source model for CNTFETs by implementing the proposed total-gate capacitance network into it, yielding a promoted version that agrees well with our measured C−V characteristics and successfully incorporates several traits associated with interface traps, such as the degradation of subthreshold swing and mobility. Besides, the proposed CNTFET capacitance model exhibits strong generality across the device architectures. The gate capacitance network topology is compatible with most common CNTFET configurations, while key parameters such as *C_OX_*, *C_Q_*, *D_it_*, and *τ* are empirically modeled from measured C−V characteristics without relying on specific device geometries. This framework enables consistent characterization of interface-trap effects in CNTFETs with varying structures and dimensions, as validated through both large-area MOSCAP measurements and scaled device analyses.

## Data Availability

Dataset available on request from the authors.
